# Seeking Health

**Published:** 1856-01

**Authors:** 


### SEEKING HEALTH.

In our June number for last year, there were some suggestions made to invalid clergymen as to the means of regaining their health without the necessity for intermitting the labors of their office. Among these were instructions for literally carrying out one of the last injunctions of THE MASTER, "*As ye go, Preach!*" A happy confirmation of the practicability of our suggestions, is found in the statement of a Missionary in the North of England. A servant girl was sent to show him the way at a particular part of his journey. On parting with her, he spoke a kindly word to her, closing with a scripture expression, "*Seek ye the Lord while he may be found*," and passed on, not expecting to meet her again, until the judgment. Some years after he did meet her, a grown woman, a widow and long a consistent Christian; on inquiry it was found, that the text which he quoted was like a nail planted in a sure place, fixed by the master of assemblies.

The clergy of this country are hard worked men, and if any class in the community ought to have some weeks recreation in summer time, they should. Without therefore dictating to them how that recreation ought to be enjoyed, we simply suggest a manner of taking it, rather different from the common method of going to the sea-shore and visiting watering places.

What does a hard working clergyman want, in the heat of mid-summer, when both mind and body are so worn and relaxed, that thought becomes an effort and a burden? Does he want to be put to sleep on the shelf of a steamboat, to occupy some pent-up room with scarcely space to turn around, to inhale the air already tainted by the natural and artificial perfumery of the five or six hundred other invalids under the same roof; or if he takes a walk, to breathe an atmosphere saturated with the clouds of dust which a thousand coming and departing vehicles, raise in the vicinity of the Hotels of New Port and Saratoga; to lounge about in semi-drowsiness for hours and hours of the day time, to be deprived of all connected slumber during the night by late coming or early going guests, as well as by the "hoppers" who do not retire until the small hours of the morning come; to be tempted beyond nature by highly seasoned food, more disease-engendering than any thing he can get at home; to guzzle down every morning, whole pints, miscalled spring-water, whose villanous smell turns a healthy stomach a mile off; and last, not least, to be kept on the strain of his proprieties, more unrelenting than among his own parishioners? Does a worn out mental worker require any of these to aid in his restoration? What infatuation of fashion! There have been times among the warring of nations, when soldiers from the able bodied were too few to meet the emergencies of the occasion, and levies had to be made among the feeble, the sick, and the aged. All reflecting men must admit that now, as in the Saviour's day, the *Harvest truly is great, but the laborers are few.* Under ordinary circumstances this is so, but it becomes a truth with an emphasis when we look at the hordes of refugees, criminal and atheistic foreigners which flock to our shores at the rate of nearly a thousand a day into the port of New York alone. We think that fact of itself makes it an emergency of the times which warrants a levying on the abilities of the sick and feeble folk of the church, for every little helps.

Our plan for clerical summer recreation is this: let every man leave his door on a good horse, with a Bible, Hymn Book and Concordance, and one change of inner clothing in his saddle-bags, let him take a direct line for any point a hundred and fifty miles west of the Mississippi, making his appointments ahead, as stump politicians and the good old methodist circuit riders used to do; a sermon for twelve o'clock and "candle lighting" at another place, of each day, at some hotel, post office, or country village, having family prayers wherever he passes a night, and leaving some kindly impressive word or phrase with almost every person with whom he is brought in contact. Can any rational mind doubt that with such a general course, a better footing up will result for health here, reflection in after life, for reward at the judgment day, than there would be from a summer at the Spa?

